# Exploiting large-scale drug-protein interaction information for computational drug repurposing

**DOI:** 10.1186/1471-2105-15-210

**Published:** 2014-06-20

**Authors:** Ruifeng Liu, Narender Singh, Gregory J Tawa, Anders Wallqvist, Jaques Reifman

**Affiliations:** 1 Department of Defense Biotechnology High Performance Computing Software Applications Institute, Telemedicine and Advanced Technology Research Center, U.S. Army Medical Research and Materiel Command, Fort Detrick, MD 21702, USA

**Keywords:** Drug repurposing, Bayes theorem, Drug-protein interaction, Mechanism of drug action

## Abstract

**Background:**

Despite increased investment in pharmaceutical research and development, fewer and fewer new drugs are entering the marketplace. This has prompted studies in repurposing existing drugs for use against diseases with unmet medical needs. A popular approach is to develop a classification model based on drugs with and without a desired therapeutic effect. For this approach to be statistically sound, it requires a large number of drugs in both classes. However, given few or no approved drugs for the diseases of highest medical urgency and interest, different strategies need to be investigated.

**Results:**

We developed a computational method termed “drug-protein interaction-based repurposing” (DPIR) that is potentially applicable to diseases with very few approved drugs. The method, based on genome-wide drug-protein interaction information and Bayesian statistics, first identifies drug-protein interactions associated with a desired therapeutic effect. Then, it uses key drug-protein interactions to score other drugs for their potential to have the same therapeutic effect.

**Conclusions:**

Detailed cross-validation studies using United States Food and Drug Administration-approved drugs for hypertension, human immunodeficiency virus, and malaria indicated that DPIR provides robust predictions. It achieves high levels of enrichment of drugs approved for a disease even with models developed based on a single drug known to treat the disease. Analysis of our model predictions also indicated that the method is potentially useful for understanding molecular mechanisms of drug action and for identifying protein targets that may potentiate the desired therapeutic effects of other drugs (combination therapies).

## Background

By conservative estimates, we know the molecular basis of more than 4,000 human diseases, whereas treatments are available for only about 250 of them [1]. The modern drug discovery paradigm, i.e., starting with a disease target and looking for a highly selective small molecule that interacts strongly only with the intended target, is struggling to meet our medical and social requirements [2]. Current estimates indicate that it takes an average of 14 years at a cost of close to $2 billion to bring a new, safe, and efficacious drug to market [3]. In the process, more than 90% of the drug candidates fail due to safety concerns, inadequate bioavailability, or lack of efficacy [3]. In reality, highly selective compounds are rare. The large number of safety and bioavailability issues facing candidate drugs, as well as reported side effects of marketed drugs, is a reflection of many undocumented and deleterious interactions between drugs and human targets. In addition, many underlying disease causes are multifactorial and can be due to dysfunctional processes involving multiple biomolecules. Even though significant efforts are devoted to understanding the molecular details of drug action, the fact is that the mechanisms of action of many efficacious drugs are poorly understood and, in many cases, remain largely unknown [4].

Drug interactions with unintended targets may lead to devastating side effects. However, these interactions may also signal the possibility for a drug to have therapeutic potential for diseases other than those for which it was approved. Indeed, there are many examples of a drug developed for a specific disease that was later approved for treating an unrelated disease [5,6]. One of the most dramatic examples is thalidomide, a drug first marketed as an anti-nausea and sedative agent prescribed to treat morning sickness in pregnant women. Thalidomide was found to cause severe birth defects and was withdrawn from the market, but it later proved to have other therapeutic effects and was approved by the United States Food and Drug Administration (FDA) for skin lesions caused by leprosy [7] and multiple myeloma [8]. In addition, thalidomide has shown promise in treating cutaneous lupus and Behcet’s disease, human immunodeficiency virus (HIV)-related mouth and throat ulcers, and blood and bone marrow cancers [9]. Although the recorded effects of thalidomide are multifaceted, with multiple underlying mechanisms possible [10], clarification of a mechanism [11] that distinguishes between the teratogenic and anticancer therapeutic effects of thalidomide [12,13] was only recently identified. The success of drug repurposing, i.e., finding new uses for existing drugs, via serendipitous discoveries inspired the development of many computational approaches for the discovery of the yet unknown therapeutic potentials of existing drugs [14-24].

A common approach used in drug repurposing is to build a binary classifier based on a training set consisting of drugs with and without a desired therapeutic effect as the positive and negative classes, respectively. One of the requirements for developing a statistically sound classifier is the availability of a relatively large number of drugs in the training set. Furthermore, it is desirable to have an equal number of drugs with and without the desired therapeutic effect in the training set so as not to bias classifier training. However, when there are many drugs approved for treating a disease, the need for discovering more drugs for the same disease is less than that for a disease for which there is a very limited number of drugs or no drugs at all. Thus, there is a need to develop computational drug repurposing methods that can be applied to diseases for which there are very few known pharmacological options.

In this article, we report on the development of a computational approach termed “drug-protein interaction-based repurposing” (DPIR) that is potentially applicable to diseases for which a very limited number of drugs are available. We based DPIR on large-scale drug-protein interaction profiles and Bayesian statistics to decipher the drug-protein interactions indicative of a desired therapeutic effect. These drug-protein interactions are then used to identify other drugs that are likely to have the same therapeutic effect.

## Methods

### Source of large-scale chemical-protein interaction information

To create large-scale chemical-protein interaction profiles for FDA-approved drugs and drug development candidates, we exploited the Search Tool for Interactions of Chemicals (STITCH) database [25]. The October 2013 release of the database (STITCH 3.1) contains chemical-protein interaction information, derived from a broad range of sources, between 300,000 small molecules and 2.6 million proteins from 1,133 organisms. The database provides a confidence measure for each chemical-protein interaction calculated by the equation *score* = 1 – Π_*i*_(1 – *p*_*i*_), with corrections that take into account the possibility of observing an interaction by chance [25]. In the equation, *p*_*i*_ denotes the confidence of interaction from the *i-*th information source. Based on STITCH, a score between 0.40 and 0.70 indicates medium confidence, between 0.70 and 0.90 indicates high confidence, and between 0.90 and 1.00 indicates the highest confidence.

To retain high-confidence chemical-protein interactions, we filtered out entries in STITCH 3.1 with confidence scores of <0.70. In addition, we removed all entries of chemical interaction with non-human proteins. The filtering reduced the total number of small molecule-protein interaction entries from >171 million to just over a half million. The categories of chemical-protein interactions with the highest occurrence in the database are binding (chemical binds to protein), inhibition (chemical inhibits protein function), and activation (chemical enhances protein function). Because the therapeutic effects of most drugs are due to chemical modulation of protein function, functional information of chemical-protein interactions, i.e., inhibition or activation, is important. However, this information is not always available. Instead, the most prevalent type of interaction information is binding. To create drug-protein interaction profiles relevant for drug repurposing, we retained interactions of only these three categories. This left 445,162 interactions between chemicals identified by 232,765 unique STITCH chemical identifiers and 6,399 unique human proteins.

### Source of FDA-approved drugs and drug development candidates

To generate a list of FDA-approved drugs and drug development candidates, we retrieved the SMILES strings of all structurally unique small molecule compounds in DrugBank [26]. Molecular structures represented by the SMILES strings were standardized, i.e., we stripped salts, standardized charge representation, removed stereochemistry labeling, removed single atom fragments, neutralized bonded zwitterions, and protonated acids/deprotonated bases. After structure standardization, we generated canonical SMILES and removed duplicates, resulting in 4,902 unique entries. They consisted of 1,163 FDA-approved drugs, 3,630 drug development candidates, 55 nutraceuticals, and 54 drugs withdrawn from market. These molecules are all referred to as “drugs” in the remainder of this article.

### Computational prediction of drug-protein interactions

Most of the compounds in DrugBank are in the biological activity screening libraries of pharmaceutical companies, government research laboratories, and academic institutions. However, not all of the DrugBank compounds have been tested in all assays evaluating chemical-protein interactions and, hence, the data collected in the STITCH database do not cover all drug-protein interactions. Thus, to create as complete drug-protein interaction profiles as possible, we complemented the drug-protein interactions contained in the STITCH database with predicted drug-protein interactions based on chemical structural similarity. This was accomplished by re-implementing the similarity ensemble approach (SEA) [27] and predicting additional drug-protein interactions based on the collection of chemical-protein interactions contained in STITCH 3.1. SEA predictions are based on two-dimensional molecular structure similarity as measured by Tanimoto coefficients between a drug molecule and all known ligands of a protein. When the similarity score is high, the probability that the drug interacts with the same protein is high. In this study, we retained drug-protein interaction predictions with a *p-*value cutoff of 0.01, and combined these predictions with the high-confidence drug-protein interactions contained in the STITCH database. The so-constructed final set of drug-protein interactions is available for non-commercial use (via download at http://www.bhsai.org/downloads/drugrepurposing/).

### Creation of a machine-readable representation of drug-protein interaction profiles

For the application of machine learning techniques, we created a binary bit-string representation of the drug-protein interaction profile for each drug. In the bit-string representation, each protein was assigned up to three bit positions to encode *1*) drug interaction (binding) with the protein, *2*) drug activation of the protein, and *3*) drug inhibition of the protein. If a drug was recorded in STITCH and/or predicted to bind to a protein, the bit representing this drug-protein interaction was turned on, i.e., assigned a value of 1 (on-bit). Otherwise, the bit was turned off (assigned a value of 0). The bits representing drug activation and drug inhibition of a protein were similarly set. To reduce memory usage, when a bit was off for all drugs, the bit was removed. The length of the final binary string was 8,769 bits, representing drug interactions with 5,516 unique human proteins. If the SEA-predicted drug-protein interactions were excluded, the length of the final binary string would be 7,886, representing interactions between 4,369 drugs and 5,003 unique human proteins. Thus, in our final drug-protein interaction profile dataset, the number of drugs whose high-confidence protein interaction information was not found in the STITCH 3.1 database, but predicted by SEA, was 533. In other words, SEA predictions contributed about 10% of the drug-protein interaction pairs in our final drug-protein interaction profiles.

### Method for identifying drug-protein interactions contributing to a desired therapeutic effect

**Figure 1:**
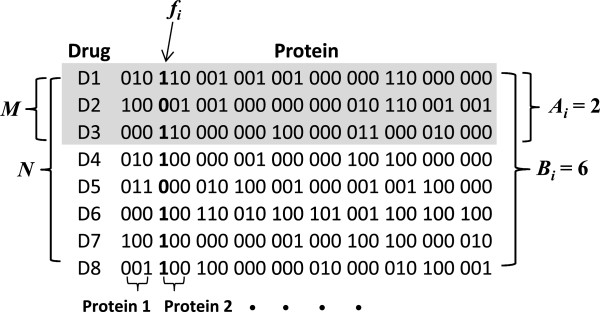
**Schematic bit-string representation of a drug-human protein interaction profile.** Each protein is represented by 3 bits to encode drug binding, drug activation, and drug inhibition of the protein, respectively. When a drug has been reported or predicted to bind, activate, or inhibit a protein, the bit representing the specific drug-protein interaction is turned on (assigned a value of 1). Otherwise, the bit is off (assigned a value of 0). *M* denotes the number of drugs with an approved indication (positive class), *N* denotes the total number of drugs, *f*_*i*_ represents on-bit or off-bit of the *i*-th bit feature, *A*_*i*_ denotes the number of on-bits of the *i*-th bit feature in the positive class, and *B*_*i*_ denotes the number of on-bits of the *i*-th bit feature in all drugs.

We found that the Laplacian-corrected Bayesian method of Xia et al. [28] was suitable for identifying drug-protein interactions indicative of a desired therapeutic effect. To illustrate this method, we used a collection of drug-protein interaction profiles, as shown in Figure 1. Given that *M* of the *N* drugs are approved for a certain disease, we assigned them to the positive class, i.e., those drugs that are known to have a desirable therapeutic effect. The number of on-bits of bit feature *f*_*i*_ among the positive class is denoted by *A*_*i*_ and the number of on-bits of the same bit feature among all drugs is denoted by *B*_*i*_. For drug repurposing, we need to estimate the conditional probability *p*(+|*f*_*i*_) of a drug-protein interaction represented by bit feature *f*_*i*_ to be responsible for the desired therapeutic effect of the drugs in the positive class. Bayes theorem gives the following:

(1) $$p\left(+|f_{i}\right)=\frac{P\;\left(+\right)\times p\left(f_{i}|+\right)}{p\left(f_{i}\right)}=\frac{\frac{M}{N}\times \frac{A_{i}}{M}}{\frac{B_{i}}{N}}=\frac{A_{i}}{B_{i}},$$

where *P*(+) is the prior probability for a drug to be positive, *p*(*f*_*i*_|+) is the conditional probability of *f*_*i*_ to be an on-bit in the positive class, and *p*(*f*_*i*_) is the probability of *f*_*i*_ to be an on-bit in all drugs. In most cases, *Eq.1* provides a reasonable estimate of *p*(+|*f*_*i*_). However, when a bit feature is severely under-sampled, such as in a situation where *B*_*i*_ = *A*_*i*_ = 1, then *p*(+|*f*_*i*_) = 1.0, which provides an overly optimistic probability estimate. However, when more samples with this bit feature on are included in the data set, *p*(+|*f*_*i*_) will most likely decrease. Thus, for diseases with few drugs, we expect the number of drugs in the positive class to be very small, and therefore undersampling of bit features may be common, leading to an inaccurate probability estimate of the predictive value of the bit feature. To correct for the effect of undersampling, one could assume that if this bit feature were sampled *K* more times, a reasonable estimate of the number of positive drugs would be *K* × *P*(+). Thus, the corrected probability is as follows:

(2) $$p_{c}\left(+|f_{i}\right)=\frac{A_{i}+\left[K\times P\left(+\right)\right]}{B_{i}+K}.$$

This correction ensures that as *B*_*i*_ → 0 and *A*_*i*_ → 0, *p* (+|*f*_*i*_) approaches the prior probability *P*(+). When *K* is set to 1/*P*(+), the adjustment corresponds to the Laplacian correction [29].

Following Xia et al. [28], we defined a weight *w*_*i*_ to represent the contribution of the *i*-th bit-feature *f*_*i*_ to the desired therapeutic effect of the positive class, as follows:

(3) $$w_{i}=\text{log}\left(\frac{p_{c}\left(+|f_{i}\right)}{P\left(+\right)}\right).$$

Once *w*_*i*_ for every *f*_*i*_ has been determined from a training set consisting of positive and negative classes of drugs, one can rank a given drug by its likelihood of having the desired therapeutic effect of the positive drug set based on the following score:

(4) $$\text{score}=\sum_{i=1}^{n}w_{i}\times f_{i},$$

where *f*_*i*_ equals 1 or 0, representing on and off bits, respectively. The higher the score a drug receives, the more likely it has the therapeutic effect of the positive class of the training set. In this formulation, the numeric score itself is never used to calculate an absolute probability. Rather, it is only used to rank the likelihood of a drug in a given disease model as a possible repurposing candidate for that disease.

## Results and discussion

### Details of model development and quality assessment

**Figure 2:**
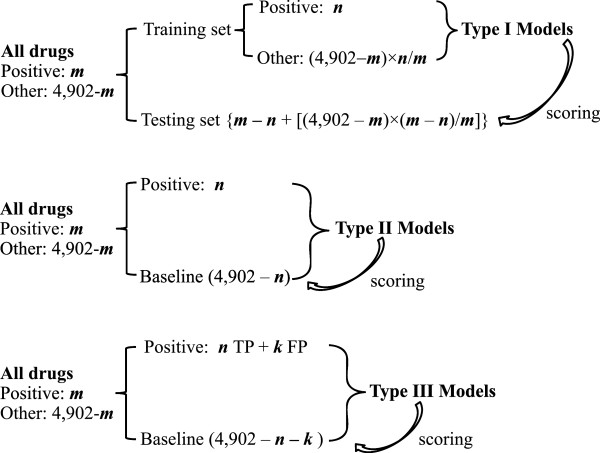
**Three machine learning approaches for developing data-driven drug repurposing models.** The total number of drugs and drug development candidates with drug-protein interaction profiles is 4,902. *m* denotes the number of drugs with a desirable therapeutic effect (positive class), *n* represents a subset of *m* used as the positive class of the training set for model development, and *k* denotes the number of drugs that do not have a desired therapeutic effect but can be used as false positives (FP) for the purpose of model development. TP: true positive.

To assess performance of the drug repurposing method described above, we used three model development procedures, as shown in Figure 2. Type I model development represents a conventional machine learning process in which a data set is segregated into a training set and a testing set. The training set consists of a subset of samples of the positive class and a subset of samples of the negative class. The remaining samples, including both positive and negative samples, are grouped into the testing set. The model parameters are determined by the training set only. The model is then applied to the testing set to assess its ability to distinguish the positive from the negative samples. In principle, type I models are not suitable for drug repurposing applications because most drugs were developed for treating a specific disease. Accordingly, for most drugs, their ability to treat other diseases has not been systematically evaluated and, in most cases, one cannot confidently label true negative drugs (samples) in the training set.

A more robust model development approach is represented by a type II model, which is trained with a subset of the positive drugs as the positive class and all other drugs collected in a baseline class, i.e., a large set of compounds that may or may not include drugs with a desired therapeutic effect. Because all drugs are used for model development, there is no testing set. However, for drug repurposing, one can simply score all the drugs assigned to the baseline class with the model and evaluate the degree of enrichment of the (known) positive drugs in the highest-scored samples. Type II models are more appropriate than type I models for drug repurposing, based on the premise that there exist drugs with yet unknown desirable therapeutic effects for a disease among the marketed drugs.

Type III models are constructed for the purpose of examining the impact of false positives on model development. In this model-building process, *k* baseline drugs (those not known to have the same therapeutic effect of the drugs of the positive class) are purposely introduced in the positive class as false positives.

### DPIR prediction analysis

To assess the performance of the proposed drug repurposing method, we performed detailed cross-validation analysis using FDA-approved drugs for hypertension, HIV, and malaria. These diseases were selected based on two criteria: the disease is clinically well defined and a significant number of FDA-approved drugs are available for cross-validation analyses. Note that our goal was to develop a method that can be applied to a disease with as few as one approved drug. However, for evaluating the performance of the DPIR method and establishing the advantages and disadvantages of different model training processes, we needed to use diseases with a significant number of approved drugs.

For hypertension, 55 single-component (non-combination) drugs on the National Institutes of Health (NIH) high blood pressure (HBP) drug list [30] have drug-protein interaction information. For HIV, 20 single-component drugs on FDA’s HIV drug list [31] have drug-protein interaction information. For malaria, 7 single-component FDA-approved drugs were listed on the Centers for Disease Control and Prevention (CDC) malaria treatment Web site [32]. In addition, we searched DrugBank and identified 4 additional drugs that were approved for treating malaria but are not in the current CDC’s list because of drug resistance, severe side effects, or because they were replaced by newer generations of antimalarial drugs of the same class. We included these 4 drugs to define a set of 11 antimalarial drugs for analysis. The names of these drugs, their molecular structures in the form of SMILES strings, and the number of on-bits in the drug-protein interaction profiles are available via publicly available download at http://www.bhsai.org/downloads/drugrepurposing. Even though the bit-length of the drug-protein interaction profiles is 8,769, for a specific drug the number of on-bits is much lower. It depends on how many proteins a drug interacts with and how many of these interactions have been identified. Thus, newer drugs tend to have a lower number of on-bits, as the synthetic routes may be under patent coverage and, therefore, the compounds are less likely to be found in the catalogs of chemical suppliers. Compounds extracted from natural products (such as some antimalarial drugs) also tend to have lower numbers of on-bits, simply because these compounds are more expensive to acquire than their synthetic counterparts and, therefore, are found in fewer screening libraries. Among the 20 HIV drugs, the number of on-bits ranges from 1 (etravirine, approved by the FDA in 2008) to 42 (ritonavir, approved by the FDA in 1996), with the average number of on-bits being 12. Among the 11 antimalarial drugs, the number of on-bits ranges from 1 (lumefantrine) to 57 (quinine) with an average of 20. Among the 55 hypertension drugs, the number of on-bits ranges from 4 (fosinopril) to 111 (verapamil) with an average of 36.

Using the drugs for each disease, we performed complete cross validation by training type I, II, and III models using one, two, or three approved drugs in the positive classes. To perform complete cross validation of models developed using a single positive drug in the positive class, each and every positive drug was used once as the positive class to build a model. For complete cross validation of models developed with two (three) positive drugs in the positive class, each unique pair (triplet) of the positive drugs was used once as the positive class to develop a model. With an increasing number of positive drugs and increasing number of positive drugs assigned to the positive class for model development, the total number of models for complete cross validation increases dramatically. For instance, with 55 HBP drugs for hypertension, complete cross validation for models developed with 3 positive drugs required training of 26,235 models. Despite the large number of models to train, the DPIR method is computationally efficient as there is no regression or optimization of any parameters required. We assessed the performance of the models by degree of enrichment of positive drugs in the top-ranked testing sets or baseline classes.

### Performance of type I models

**Figure 3:**
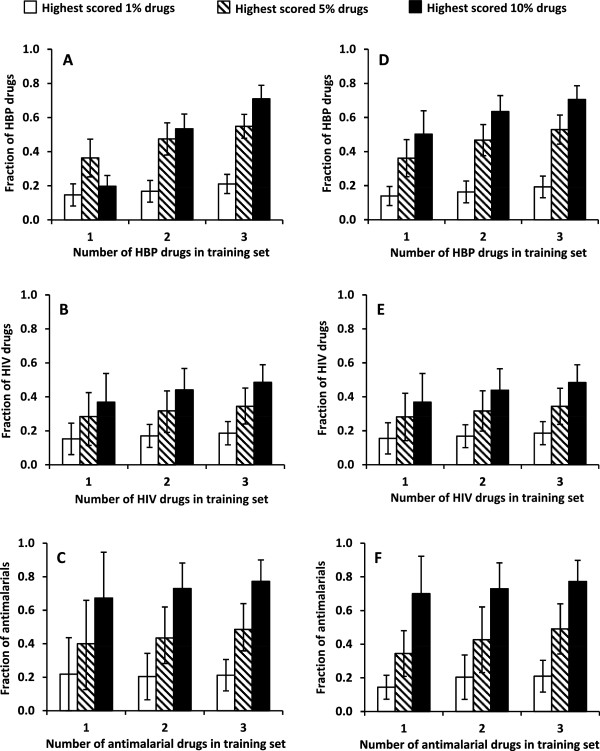
**Performance comparison between type I and type II models.** Comparison of enrichment efficiencies of type I **(A-C)** and type II **(D-F)** models for high blood pressure (HBP), HIV, and antimalarial drugs. The models were built with one, two, and three drugs in the positive class of the training set. Bar heights denote the fraction of FDA-approved HBP, HIV, and antimalarial drugs in the testing set (type I models) or baseline class (type II models) that scored in the highest 1%, 5%, and 10% of the compounds, respectively. Error bars represent 1 standard deviation from full cross-validation calculations.

Figure 3A-C shows degrees of enrichment of positive drugs by type I models for hypertension, HIV, and antimalarial drugs. These results show that with models developed using a single positive drug in the positive class of the training set, on average, between 15% and 20% of positive drugs in the testing sets were in the highest-scored 1% of the tested samples, between 30% and 40% of positive drugs were in the highest-scored 5% of the tested samples, and between 40% and 60% of positive drugs were in the highest-scored 10% of the tested samples. The corresponding enrichments by models developed from two or three positive drugs in the positive class of the training sets were slightly higher, but, more importantly, they exhibited lower variability, as indicated by decreasing standard deviations of the fraction of positive drugs in the highest-scored testing samples. This is because increasing the sample size (number of positive drugs in the positive class) increases the signal-to-noise ratio, which improves Bayesian statistics.

The expected fractions of positive drugs randomly distributed in the testing sets were 1%, 5%, and 10% in the 1%, 5%, and 10% of testing samples, respectively. Compared with a random selection, type I models achieved significant enrichment, especially considering the small number of positive drugs (one, two, or three) present in the positive class for model development. The corresponding *p*-values for achieving such levels of enrichment by chance ranged from 6.5 × 10^-3^ to 1.8 × 10^-28^.

### Performance of type II models

Figure 3D-F shows the results of type II models for the three diseases. Even though a large number of positive drugs for each disease are in the baseline class and play the role of false negatives, they had little impact on model performance. The bar heights of type I and type II models for each disease and the standard deviations were nearly identical. The lack of appreciable impact of the false negatives was at first surprising. However, as the number of false negatives was very small compared with the total number of compounds in the various baseline classes, their effect was minimal. As a result, the false negatives had negligible impact on the weight *w*_*i*_ associated with each drug-protein interaction. Thus, the results shown in Figure 3 indicate that false negatives have a negligible impact on the model development.

### Impact of false positives on model performance

**Figure 4:**
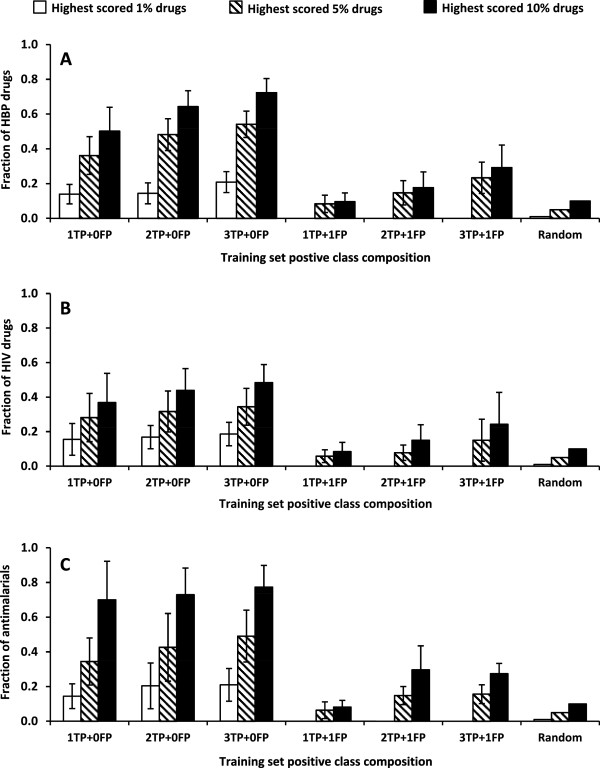
**Impact of false positive on the performance of type II models.** The models were built with one to three true positives and either zero or one false positive. Error bars represent 1 standard deviation from full cross-validation calculations. The random bar heights represent the expected fractions of positive drugs in 1%, 5%, and 10% randomly picked baseline compounds. Models constructed with no false positives correspond to type II models (Figure 3,D-F). **A**: High blood pressure (HBP) model. **B**: Human immunodeficiency virus (HIV) model. **C**: antimalarial model. TP: true positive. FP: false positive.

Figure 4 shows the comparison between type II and type III models. To develop the type III models, we included one false positive drug in the positive class for model building. The comparison shows that a false positive in the training set had a significant negative impact on model quality. Models developed with one true positive and one false positive in the positive class performed no better than picking the drugs randomly. Increasing the number of true positives in the positive class improved model performance; the improvement was not very significant as the enrichment rates of type III models were significantly lower than the corresponding enrichment rates of type II models. The practical implication is that the drugs used for the positive class in the model development should truly be associated with the desired therapeutic effect in order for the repurposing method to work efficiently.

### Model performance without SEA-predicted drug-protein interactions

To examine the impact of SEA-predicted drug-protein interactions on model performance, we re-created drug-protein interaction profiles using information only contained in the STITCH database and used the profiles to repeat the cross-validation calculations of type II models. Table 1 shows the results in comparison with those obtained using drug-protein interaction information from the STITCH database augmented by SEA predictions. For the hypertension and HIV models, the enrichment rates obtained with and without SEA-predicted drug-protein interactions were very close, with a slight increase in retrieved drugs when adding the SEA predictions. For malaria, however, the models that did not use SEA-predicted drug-protein interactions performed significantly better. The primary reason for this was that 6 out of the 11 malaria drugs shared the same mechanism of action. They consisted of quinine, a natural product extracted from the bark of cinchona trees, and 5 synthetic analogs of quinine: amodiaquine, mefloquine, chloroquine, hydroxychloroquine, and primaquine. Because they shared the same mechanism of action, this mechanism of action was already well represented by the drug-protein interactions in the STITCH database. Thus, for this model, the additional SEA-predicted interactions introduced mechanistic noise through irrelevant interactions that led to a degraded model performance.

**Table 1:** **Impact of SEA-predicted drug-protein interactions on Type II model performance**^**a**^

		**With SEA**		**Without SEA**	
**Evaluation of top-ranking scores (%)**	**Drugs in training set**	**Fraction**	**σ**	**Fraction**	**σ**
**High Blood Pressure model**					
1	1	0.14	0.06	0.15	0.07
5	1	0.36	0.11	0.36	0.11
10	1	0.50	0.14	0.48	0.14
1	2	0.14	0.06	0.18	0.07
5	2	0.48	0.09	0.45	0.10
10	2	0.64	0.09	0.60	0.11
1	3	0.19	0.06	0.20	0.07
5	3	0.53	0.08	0.52	0.10
10	3	0.70	0.08	0.67	0.10
**HIV model**					
1	1	0.16	0.09	0.20	0.19
5	1	0.28	0.14	0.30	0.18
10	1	0.37	0.17	0.39	0.21
1	2	0.17	0.07	0.18	0.07
5	2	0.32	0.12	0.33	0.10
10	2	0.44	0.13	0.43	0.15
1	3	0.19	0.07	0.21	0.07
5	3	0.34	0.11	0.37	0.09
10	3	0.48	0.10	0.50	0.12
**Malaria model**					
1	1	0.14	0.07	0.20	0.24
5	1	0.34	0.14	0.54	0.25
10	1	0.70	0.22	0.79	0.22
1	2	0.20	0.13	0.20	0.13
5	2	0.43	0.20	0.58	0.16
10	2	0.73	0.15	0.89	0.10
1	3	0.21	0.09	0.21	0.10
5	3	0.49	0.15	0.66	0.13
10	3	0.77	0.13	0.91	0.07

^a^The type II models were built with one, two, and three positive drugs in the positive class of the training set. The fraction of positive drugs in the baseline class that scored in the highest 1%, 5%, and 10% of the compounds are recorded for models using the STITCH 3.1 database with and without SEA-predicted drug-protein interactions. Fraction, fraction of known drugs retrieved; σ, standard deviation; SEA, similarity ensemble approach.

In summary, the main contribution of the SEA predictions was to make ~10% of the drugs whose protein interaction information was not available in STITCH 3.1 amenable to repurposing. As an example, among the 20 HIV drugs, high-confidence drug-protein interaction information for fosamprenavir, a pro-drug, was not present in STITCH 3.1. Therefore, this compound would not be amenable to repurposing by DPIR without including the SEA-predicted interactions. The additional 11 protein interactions with fosamprenavir, derived from the SEA predictions, allowed us to include it in our modeling analyses.

### Impact of increasing number of drugs in the positive class on model performance

In the above analyses, all models were built with one to three positive drugs to simulate the situation of a disease with very few approved drugs. To evaluate the impact of increasing the number of positive drugs on model performance, we also performed cross-validation calculations using 1, 5, 11, and 54 hypertension drugs in the positive class for type II model development, and we evaluated the enrichment power of the resulting models for the hypertension drugs left in the baseline class. On average, among the highest-scored 1% baseline compounds, there were 13%, 25%, 33%, and 56% hypertension drugs with models developed with 1, 5, 11, and 54 hypertension drugs. The corresponding hypertension drugs in the highest-scored 5% baseline compounds were 36%, 61%, 71%, and 85%, respectively, and the corresponding hypertension drugs in the highest-scored 10% baseline compounds were 50%, 77%, 85%, and 100%. We obtained similar results from the other models (data not shown). Thus, as expected, increasing the number of compounds in the training set increased the number of positive drugs retrieved.

### Pharmacological information of top-scored baseline drugs

The analyses above indicate that models derived by increasing the size of the positive class have increasingly better performance. In practical applications, one should include all drugs approved for an indication/disease in the positive class of the training process. To this end, we developed our final models for hypertension, HIV, and malaria by including all the drugs for each disease in the respective positive class. We used the resulting type II models to score the baseline compounds and we assessed the predictive power of the models by performing literature searches of the relevant information for the 10 highest-scored FDA-approved drugs for each disease. Tables 2, 3 and 4 summarize the results.

**Table 2:** **Therapeutic information of the drugs assigned to the baseline class that were scored highest by the hypertension model**^**a**^

**Generic name**	**Score**	**Information from DrugBank**^**b**^	**Information from other sources**
Nitrendipine	103.9	For mild to moderate *hypertension.*	
Nimodipine	93.9	For use as an adjunct to improve neurologic outcome following subarachnoid hemorrhage.	A 1998 clinical study [33] observed that nimodipine reduces systolic and diastolic blood pressures of the central retinal artery and therefore may provide another therapeutic choice for pregnancy-induced hypertension.
Alprenolol	69.7	For *hypertension,* angina, and arrhythmia.	
Nilvadipine	69.2	For vasospastic angina, chronic stable angina, and *hypertension.*	
Oxprenolol	69.1	For the treatment of *hypertension*, angina pectoris, arrhythmias, and anxiety.	
Norepinephrine	53.8	For patients in vasodilatory shock states, also used as a vasopressor medication for patients with critical *hypotension.*	
Spirapril	52.1	An ACE inhibitor for *hypertension.*	
Lercanidipine	46.5	For *hypertension*, angina pectoris, and Raynaud’s syndrome.	
Yohimbine	44.7	Used as a mydriatic and for the treatment of *impotence.*	
Epinephrine	43.9	Used in asthma and cardiac failure and to delay absorption of local anesthetics.	A systematic review in 2002 [34] found that the use of epinephrine in uncontrolled hypertensive patients was associated with small, non-significant increases in systolic and diastolic blood pressure.

^a^The model was developed with all 55 drugs in the National Institutes of Health hypertension drug list in the positive class; the remaining drugs and drug development candidates were potential repurposing candidates in the baseline class.

^b^Ref. [26].

ACE: angiotensin-converting enzyme.

**Table 3:** **Therapeutic information of the drugs assigned to the baseline class that were scored highest by the HIV model**^**a**^

**Generic name**	**Score**	**Information from DrugBank**^**b**^	**Information from other sources**
Verapamil	23.5	For hypertension, angina, and cluster headache prophylaxis.	A 1991 study [37] observed that verapamil at high concentration can potentiate HIV-1 replication in lymphoid cells.
Clotrimazole	22.6	For oropharyngeal candidiasis, vaginal yeast infections, and fungal infections.	
Ketoconazole	21.5	For fungal infections.	
Dexamethasone	21.0	An anti-inflammatory 9-fluoro-glucocorticoid.	A 2001 study [36] found that dexamethasone inhibits CD4 T cell death mediated by macrophages from HIV-infected persons.
Amprenavir	19.6	For treatment of *HIV-1 infection* in combination with other antiretroviral agents.	
Atorvastatin	19.3	For hypercholesterolemia.	A 2004 study [35] concluded that statins inhibit HIV-1 infection by downregulating Rho activity.
Clarithromycin	18.5	Antibiotic.	
Lovastatin	18.2	For hypercholesterolemia.	A 2004 study [35] concluded that statins inhibit HIV-1 infection by downregulating Rho activity.
Quinidine barbiturate	18.1	For the treatment of ventricular pre-excitation and cardiac dysrhythmias.	
Cimetidine	17.9	For acid-reflux disorders (GERD), peptic ulcer disease, heartburn, and acid indigestion.	

^a^The model was developed with all 20 HIV drugs in the FDA’s HIV drug list in the positive class; the remaining compounds were potential repurposing candidates in the baseline class.

^b^Ref. [26].

**Table 4:** **Therapeutic information of the drugs assigned to the baseline class that were scored highest by the malaria model**^**a**^

**Generic name**	**Score**	**Information from DrugBank**^**b**^	**Information from other sources**
Dexamethasone	22.1	An anti-inflammatory 9-fluoro-glucocorticoid.	Dexamethasone was reported to have a dramatic life-saving effect on people with cerebral malaria [44]. However, two subsequent placebo-controlled clinical trials failed to demonstrate clinical benefit [45,46].
Verapamil	21.5	A calcium channel blocker for hypertension, angina, and cluster headache prophylaxis.	A 1995 study [48] reported that verapamil reverses chloroquine resistance in the malaria parasite.
Quercetin	18.9	A flavonol found in plants, antioxidant.	A 2012 study [47] reported that quercetin had antiplasmodial activity.
Miconazole	16.5	An imidazole antifungal agent.	Many studies [39-43] have reported antimalarial activities of antifungal azole compounds.
Clotrimazole	15.9	An imidazole derivative with a broad spectrum of antimycotic activity.	Many studies [39-43] have reported antimalarial activities of azoles, including clotrimazole [43].
Cimetidine	15.5	For acid-reflux disorders (GERD), peptic ulcer disease, heartburn, and acid indigestion.	A 1997 study [49] reported synergism of cimetidine with antimalarial agents. It is ineffective when used alone.
Ketoconazole	15.2	For systemic fungal infections.	Many studies [39-43] have reported antimalarial activities of azoles, including ketoconazole [41].
Nifedipine	14.9	A calcium channel blocker for angina, hypertension, and Raynaud's phenomenon.	
Tamoxifen	14.4	For breast cancer.	
Clobetasol	14.4	For corticosteroid-responsive dermatoses of the scalp.	

^a^The model was developed with all 11 antimalarial drugs in the positive class; the remaining compounds were potential repurposing candidates in the baseline class.

^b^Ref. [26].

Because we included all the drugs from the NIH’s hypertension drug list in the positive class for model development, the baseline class did not include any of these drugs. As shown in Table 2, among the 10 highest-scored FDA-approved drugs in the baseline class, six of them have hypertension as part of their approved indications based on information contained in DrugBank [26]. In addition, a 1998 clinical study observed that nimodipine, the second highest-scored baseline drug, reduced systolic and diastolic blood pressures of the central retinal artery, and it therefore may be a candidate for pregnancy-induced hypertension therapy [33]. However, information from DrugBank indicates that norepinephrine, the sixth highest-scored drug in Table 2, has been approved for the treatment of critical hypotension, and yohimbine was approved for the treatment of impotence. In addition, epinephrine was approved for asthma and cardiac failure, and a 2002 review found that use of this drug was associated with small non-significant increases in systolic and diastolic blood pressure [34]. It seems that these three drugs induce a blood pressure increase, contrary to the expected therapeutic effect of hypertension drugs. This apparent contradiction is rationalized because functional information for many drug-protein interactions contained in the STITCH database is incomplete. Although these drugs and the hypertension drugs interact with the same proteins, they induce an opposite functional outcome, i.e., activation instead of inhibition of the targeted proteins. Because of the lack of specific functional information, drugs interacting with the same proteins annotated as “binding” in the STITCH database were scored high by the algorithm, even though they may have opposite therapeutic effects. We similarly observed the same effect in the highest-scored baseline drugs identified by the HIV and malarial models discussed below.

Table 3 shows the results for the 10 baseline drugs scored highest by the HIV type II model. Among them, amprenavir was approved by the FDA as a treatment for HIV-1 infection in combination therapy with other anti-HIV drugs, even though it is not approved as a monotherapy for HIV. Surprisingly, two statins, atorvastatin and lovastatin, scored among the top-10 drugs, suggesting that they affect proteins that are also targeted by anti-HIV drugs. Indeed, our literature search found a 2004 study that concluded that statins inhibit HIV-1 infection by downregulating Rho activity [35]. The anti-inflammation drug dexamethasone also scored high. Our search identified a 2001 study that found that dexamethasone inhibits CD4 T cell death mediated by macrophages from HIV-infected persons [36]. Toward the end of an HIV infection, the number of functional CD4 T cells falls, which leads to the symptomatic stage of AIDS. The observation that dexamethasone inhibits CD4 T cell death supports the predictive power of our model. However, our search for the highest-scored baseline drug, verapamil, did not find a convincing literature report for its putative anti-HIV activity. Instead, a 1991 study observed that, in high concentration, verapamil can potentiate HIV-1 replication in lymphoid cells [37]. Its interaction with nuclear factor κ-light-chain enhancer of activated B cells (NF-κB) was suggested to be responsible for this effect. NF-κB was later considered a potential target of anti-HIV chemotherapy by Pande and Ramos [38]. The high scoring of verapamil in our model seems to support NF-κB as a target for anti-HIV drugs. The reason we misidentified the therapeutic effect of verapamil may again be partly rationalized by the lack of functional information (activation or inhibition) of drug-NF-κB interaction of the anti-HIV drugs in the STITCH database.

Table 4 shows the 10 drugs in the baseline class that scored highest by the antimalarial model. None of them has been approved by the FDA for the treatment of malaria. However, three of them are azole compounds (miconazole, clotrimazole, and ketoconazole), and many studies that have reported antimalarial activities of azole compounds [39-43] lend support to our model predictions. The highest-scored non-malarial drug is dexamethasone. It has been reported to have dramatic life-saving effects in people with cerebral malaria [44]. However, these effects were not observed in subsequent placebo-controlled clinical trials [45,46]. A flavonol nutraceutical, quercetin, scored third-highest, as shown in Table 3. It was reported in 2002 to have antiplasmodial activity [47], corroborating our model prediction.

It is interesting to note that two other drugs shown in Table 4, verapamil and cimetidine, do not have antimalarial activities themselves but exhibit synergism when used in combination with antimalarial drugs [48,49]. In the case of verapamil, it was reported that it reverses chloroquine resistance in the malaria parasite [48]. To understand why these two drugs scored high, we investigated the most frequently occurring drug-protein interactions among the antimalarial drugs. Among the 11 antimalarial drugs, 10 are cytochrome *P*-450 (CYP)2D6 inhibitors, 4 are CYP1A2 inhibitors, 4 are CYP3A4 inhibitors, 3 are CYP2C9 inhibitors, and 3 are CYP1A1 activators. Because of their high frequencies among the antimalarial drugs, according to *Eq.3*, the drug-protein interactions contributing most to the antimalarial model relate to the inhibition of CYP proteins. However, the human CYP proteins are unlikely malarial drug targets. Instead, the similarity between the CYP-binding site, the heme group, and malarial drug target may be the reason for the apparent importance of CYP inhibition for antimalarial activity. Indeed, one of the proposed antimalarial mechanisms of 4-amino quinolones is the formation of a drug-heme complex that inhibits the polymerization and crystallization of free heme into hemozoin [50]. The malaria parasite digests hemoglobin and produces free heme, which is toxic to the parasite. The formation of hemozoin is a parasite detoxification pathway. Quinolones with fused bicyclic aromatic rings bind well with heme due to π-π stacking interactions and therefore inhibit hemozoin efficiently. Because heme is also the most important structural moiety of the CYP enzymes, the high affinity of quinolones to heme also makes them inhibitors of CYP enzymes. Verapamil and cimetidine were scored high by the antimalarial model because both of them are inhibitors of CYP2D6, CYP3A4, and CYP2C9. As the compounds do not have a fused bicyclic aromatic moiety, they do not bind well with free heme and, hence, do not have antimalarial activity by themselves. The reason they enhance the activity of antimalarial drugs is more likely a direct effect of their inhibition of Pgh1, an ATP-binding cassette protein functioning as a membrane efflux pump [51]. It reduces antimalarial drugs in the parasite food vacuole and therefore results in drug resistance. The human homologue of Pgh1 is P-glycoprotein, a well-known multidrug-resistant protein. Both verapamil and cimetidine are inhibitors of P-glycoprotein and, therefore, are also likely inhibitors of Pgh1; thus, co-administration of these drugs with traditional malarial drugs should result in enhanced antimalarial activity.

It is interesting to note that five of the ten entries in Tables 3 and 4 are overlapping. This was partly a reflection of the five drugs having an above-average number of on-bits in their protein interaction profiles, effectively leading to more off-target effects, including a heightened potential for therapeutic repurposing. The numbers of on-bits in the drug-protein interaction profiles were 445, 111, 65, 44, and 40 for dexamethasone, verapamil, cimetidine, ketoconazole, and clotrimazole, respectively, whereas the average number of on-bits of all 4,902 compounds was 38.

In summary, the results shown in Tables 2, 3 and 4 support the conclusions we derived from the results shown in Figures 2 and 3: that the performance of the drug-repurposing method developed in this project is robust and may have practical utility. The high-scoring drugs derived for each disease not only included drugs approved for the disease but also molecules identified in the literature as potential drugs against the disease. Thus, we can propose top-scored drugs with no recorded indication for the disease as novel drug repurposing candidates for the disease. In addition, the methodology can be used to characterize and decipher the mode of drug action and identify protein targets that may enhance the effect of existing drugs via synergistic drug combinations.

### Application to a condition with only one approved drug

Impaired blood clotting may lead to hemorrhagic shock and contribute to fatalities resulting from traumatic injuries. Currently, the only drug approved by the FDA for reducing or preventing hemorrhage in trauma is tranexamic acid. We constructed a type II model using tranexamic acid as the only drug in the positive class. We used the model to score all other compounds, and Table 5 shows the highest-scoring compounds. The highest-scored baseline compound was aminocaproic acid, a drug for the treatment of excessive postoperative bleeding [52]. As an acyclic analog of tranexamic acid, aminocaproic acid and tranexamic acid have a high level of structural similarity. Therefore, it can be expected that they have similar therapeutic effects.

**Table 5:** **Structures and therapeutic information of tranexamic acid and the highest-scored drugs by the tranexamic acid model**^**a**^

**Structure**	**Generic name**	**Score**	**DrugBank indication**^**b**^	**Information from other sources**
	Tranexamic Acid		For preventing hemorrhage in trauma and for excessive bleeding during and following tooth extraction, surgery, and menstruation.	The only drug used to build the DPIR prediction model.
	Aminocaproic acid	1.39	For the treatment of excessive postoperative bleeding.	
	Amiloride	1.38	For use as adjunctive treatment with thiazide diuretics or other kaliuretic-diuretic agents in congestive heart failure or hypertension.	Amiloride was evaluated as a treatment for ameliorating trauma-hemorrhagic shock-induced lung injury in rats [53].
… 26 experimental drugs with scores between 1.38 and 0.69 ….				
	Diethylstilbestrol	0.69	For treatment of prostate cancer and prevention of miscarriage or premature delivery in pregnant women prone to miscarriage or premature delivery.	Diethylstilbestrol was found to have particular clinical value in the treatment of certain functional gynecic aberrations. One of these is excessive or prolonged functional uterine bleeding [54].

^a^The model was developed with tranexamic acid as the only member of the positive class; all other compounds were in the baseline class.

^b^Ref. [26].

Two other marketed drugs that ranked high were amiloride, approved for congestive heart failure and hypertension, and diethylstilbestrol, approved for the treatment of prostate cancer and the prevention of miscarriage or premature delivery. Amiloride was evaluated for trauma-hemorrhagic shock-induced lung injury in a rat model [53], and diethylstilbestrol was studied for hemostasis in functional uterine hemorrhage and concluded to be of clinical value for excessive or prolonged functional uterine bleeding [54]. These observations are in line with the model predictions.

### Limitations on comparison with other methods

The comparison of different computational drug repurposing methods is limited by a lack of publicly available datasets and software systems. Further complications arise because different methods rely on different types of information, ranging from experimentally/clinically derived data (such as drug- and disease-induced gene expression changes, drug side effects, and drug-protein interactions) to calculated measures (such as drug-drug structure similarity, drug target protein sequence similarity, and semantic similarity of disease phenotypes). Thus, because most publications do not make software, training data, and test data containing all types of information available, there is little scope for proper methodological comparison, i.e., method A versus method B on the same dataset using a generally accepted standard. To facilitate future comparison with other methods, we have made our drug-protein interaction dataset publicly available for download (see Methods).

To enable a reference comparison, we compared the performance of the type II models with that of a chemical fingerprint similarity search on the same datasets. Thus, we performed cross-validation calculations using one, two, and three positive drugs as the positive classes, and the remaining compounds, including other positive drugs, as the test compounds. We used the Tanimoto chemical similarity between a test compound and a positive class compound, calculated by using Accelrys ECFP_4 fingerprints, to rank order the test compounds. Table 6 shows the calculated percentages of the positive drugs in the highest-ranked 1%, 5%, and 10% of the test set compounds for all three repurposing models. The chemical fingerprint searches provided significant enrichments, the success of which was largely determined by the presence or absence of chemically similar drugs in the approved drug set for each disease. For the hypertension and HIV drugs, the DPIR method consistently performed better than the structural similarity search. This was because the drugs used to construct the models were different from each other and structural similarity was limited, whereas the DPIR methodology, based on capturing drug-protein interactions, did not *require* structural similarities. In contrast, for malaria drugs the situation was reversed, with structural similarity being the most “successful” repurposing strategy. However, this was primarily because 5 of the 11 malaria drugs (~50%) were structurally very closely related to quinine, the original malaria drug. They (amodiaquine, mefloquine, chloroquine, hydroxychloroquine, and primaquine) are synthetic analogs of quinine and therefore structurally very similar to it. Molecular structures of the hypertension, HIV, and malaria drugs are given in Additional file 1: Figures S1, Additional file 2: Figure S2, Additional file 3: Figure S3. They show that all malaria drugs can be grouped in one structural similarity cluster of six compounds and five singletons. The hypertension drugs belong to seven structural clusters and 27 singletons, and the HIV drugs belong to six clusters and seven singletons. Thus, the hypertension and HIV drug sets represent a more structurally diverse ensemble of drugs than the malaria drugs.

**Table 6:** **Comparison of DPIR and chemical fingerprint (similarity) search-based drug repurposing approaches**^**a**^

		**DPIR**		**Similarity**	
**Evaluation of top-ranking scores (%)**	**Drugs in training set**	**Fraction**	**σ**	**Fraction**	**σ**
**High Blood Pressure model**					
1	1	**0.14**	0.06	0.09	0.07
5	1	**0.36**	0.11	0.16	0.07
10	1	**0.50**	0.14	0.24	0.10
1	2	**0.14**	0.06	0.14	0.08
5	2	**0.48**	0.09	0.22	0.09
10	2	**0.64**	0.09	0.29	0.10
1	3	**0.19**	0.06	0.18	0.08
5	3	**0.53**	0.08	0.27	0.10
10	3	**0.70**	0.08	0.33	0.10
**HIV model**					
1	1	**0.16**	0.09	0.08	0.07
5	1	**0.28**	0.14	0.20	0.09
10	1	**0.37**	0.17	0.25	0.10
1	2	**0.17**	0.07	0.11	0.07
5	2	**0.32**	0.12	0.28	0.12
10	2	**0.44**	0.13	0.36	0.12
1	3	**0.19**	0.07	0.13	0.07
5	3	**0.34**	0.11	0.32	0.13
10	3	**0.48**	0.10	0.44	0.14
**Malaria model**					
1	1	0.14	0.07	**0.26**	0.18
5	1	0.34	0.14	**0.50**	0.21
10	1	**0.70**	0.22	0.55	0.23
1	2	0.20	0.13	**0.35**	0.16
5	2	0.43	0.20	**0.62**	0.16
10	2	**0.73**	0.15	0.72	0.16
1	3	0.21	0.09	**0.40**	0.14
5	3	0.49	0.15	**0.68**	0.14
10	3	0.77	0.13	**0.79**	0.12

^a^The DPIR (type II) models were developed with one, two, or three positive drugs in the positive class of the training set. The chemical fingerprint search used Tanimoto similarity (TS) calculated with the Accelrys ECFP_4 fingerprint. The highest TS coefficient between a baseline compound and a positive compound was used to rank order the baseline compound. DPIR, drug-protein interaction-based repurposing; fraction, fraction of known drugs retrieved; σ, standard deviation; bold font, highest fraction retrieved.

In effect, DPIR could identify compounds whose activity was due to structurally similar compounds having similar activity, as well as compounds containing different structural scaffolds but having similar protein interaction profiles. This was also corroborated by the data shown in Table 5, where even though amiloride and diethylstilbestrol were structurally very different from tranexamic acid, DPIR successfully scored them high as potential repurposing candidates.

## Conclusions

In this article, we described the development of a Bayesian statistics-based computational drug repurposing method termed DPIR and assessed its performance. We demonstrated that the method required very few known drugs to build a successful predictive model for test cases for which there are many approved drugs. We also demonstrated that for trauma-induced hemorrhage, for which only one FDA-approved drug is available, the method gave high scores to two drugs approved for unrelated indications, but with potential therapeutic effects against hemorrhage as supported by literature reports. These results indicate that DPIR is potentially applicable to diseases with as few as one approved drug, a challenging situation for methods based on a binary classifier approach. DPIR relies on large-scale drug-protein interaction information. In principle, if one knows the molecular mechanisms of a disease and the details of drug-protein interactions, one can predict whether a drug will have the desired therapeutic effect for a specific disease. However, details of molecular mechanisms of drug action are not well understood and even unknown for many efficacious drugs, complicated by the fact that most drugs interact with a large number of proteins. Bayesian statistics provide a powerful and unbiased approach to identify specific drug-protein interactions critical for a desired therapeutic effect.

The DPIR method relies on the association of drug-protein interactions to capture and define the therapeutic effects of the drug based on the knowledge of at least one known drug. The underlying assumption in this approach is that the main effect of the drug is mediated through drug-protein interactions. These interactions create a characteristic profile, or signature, that can be used to find other drugs without direct knowledge of how these interactions contribute to disease treatment. Furthermore, as found in the case studies above, the true effect of the drug-protein interaction determines the therapeutic potential and cannot be predicted without accurate interaction annotations. Thus, if the drug-protein activation or inhibition effect is only annotated as “binding”, the model cannot resolve the true nature of the interaction. Experimental verification will, of course, always be required for probing the repurposing potential of any drug.

Because of the large number of druggable targets that can be associated with human diseases, a major limitation of our method is availability of large-scale drug-protein interaction information. This is not a problem for drugs that have been on the market for a long time, as these compounds are increasingly likely to be included in different screening libraries and tested in multiple bioassays over time. However, for newer drugs, information of their interactions with a broad spectrum of proteins is usually lacking and therefore may limit the applicability of the method. Yet with time, these compounds will be tested in more assays, and information about their interaction with human proteins will become available. As more and more screening results are deposited in the public domain, the aggregated data will become an invaluable resource for establishing whole genome-wide drug-protein interaction profiles and for discovering new uses for existing drugs.

## Competing interests

The authors declare that they have no competing interests.

## Authors’ contributions

All authors contributed to the design of the research. RL performed the computational implementations and analyses. RL and AW wrote the article, which was edited by JR. All authors read and approved the final manuscript.

## Supplementary Material

Additional file 1: Figure S1 Hypertension drugs grouped by molecular structure similarity. Molecular structure similarity clusters of the hypertension drugs. Click here for file

Additional file 2: Figure S2 HIV drugs grouped by molecular structure similarity. Molecular structure similarity clusters of the HIV drugs. Click here for file

Additional file 3: Figure S3 Malaria drugs grouped by molecular structure similarity. Molecular structure similarity clusters of the malaria drugs. Click here for file
