# Supplementary material for: Exploiting large-scale drug-protein interaction information for computational drug repurposing
Source: BMC Bioinformatics. 2014 Jun 20;15:210. doi: 10.1186/1471-2105-15-210 (PMC4079911; doi:10.1186/1471-2105-15-210)
Supplement: Additional file 1: Figure S1 — Hypertension drugs grouped by molecular structure similarity. Molecular structure similarity clusters of the hypertension drugs. [file 1471-2105-15-210-S1.pdf]

Figure S1. Hypertension drugs grouped by molecular structure similarity

SimilarityGroup: 1

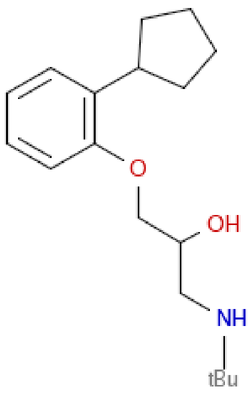

Penbutolol

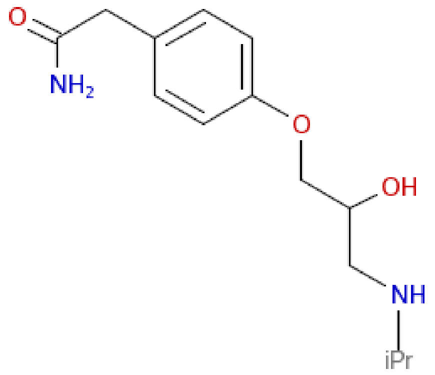

Atenolol

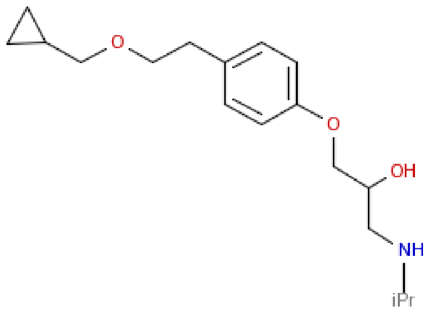

Betaxolol

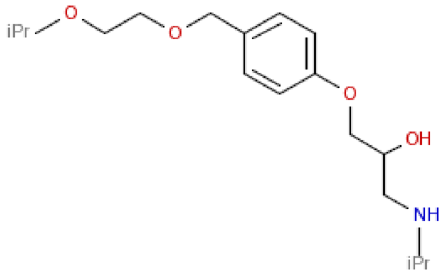

Bisoprolol

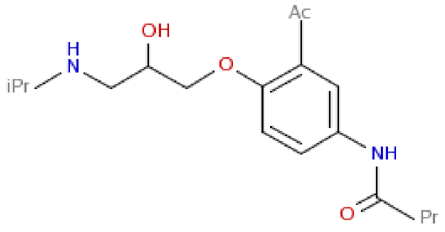

Acebutolol

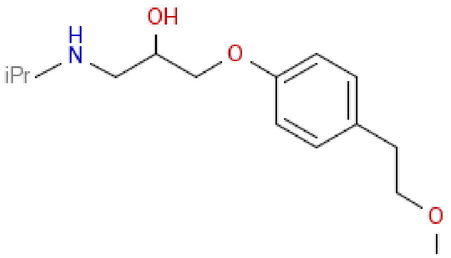

Metoprolol

SimilarityGroup: 2

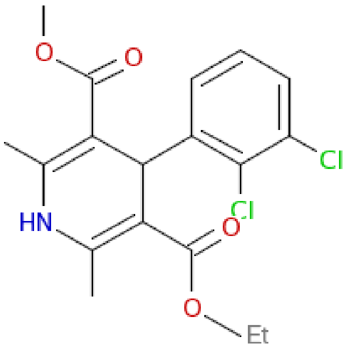

Felodipine

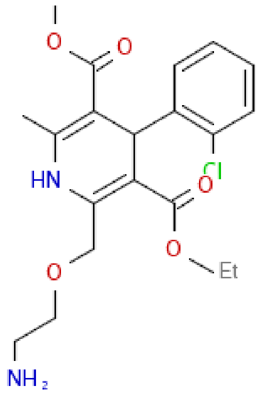

Amlodipine

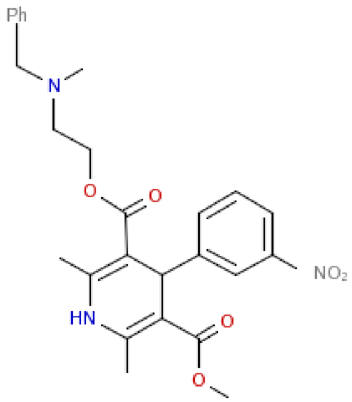

Nicardipine

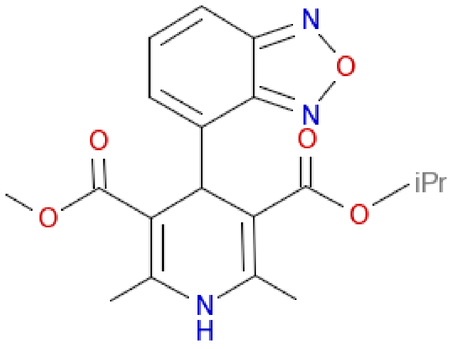

Isradipine

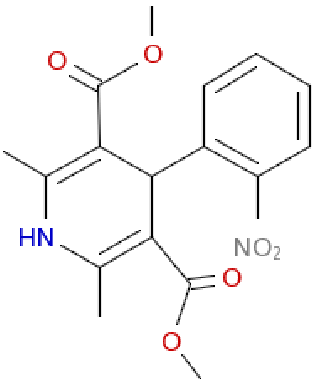

Nifedipine

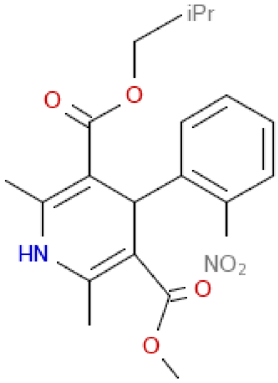

Nisoldipine

SimilarityGroup: 3

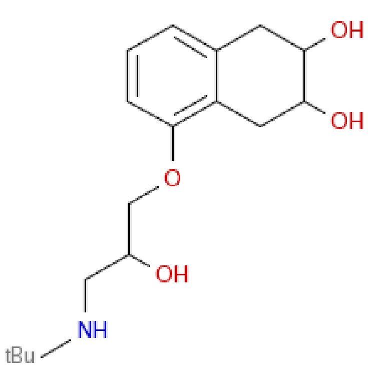

Nadolol

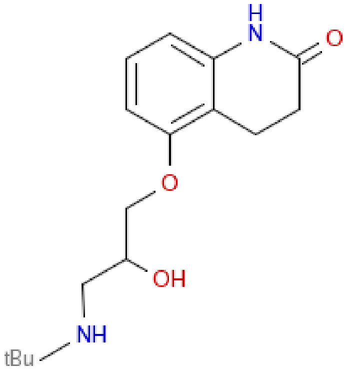

Carteolol

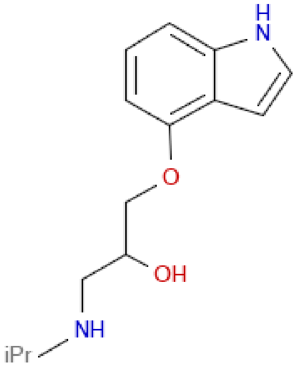

Pindolol

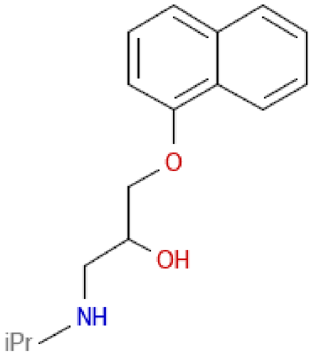

Propranolol

SimilarityGroup: 4

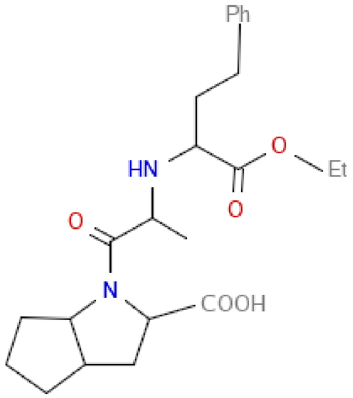

Ramipril

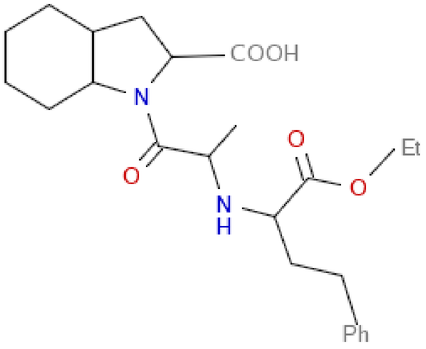

Trandolapril

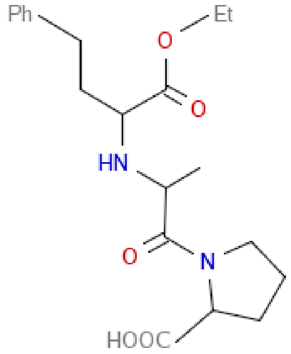

Enalapril

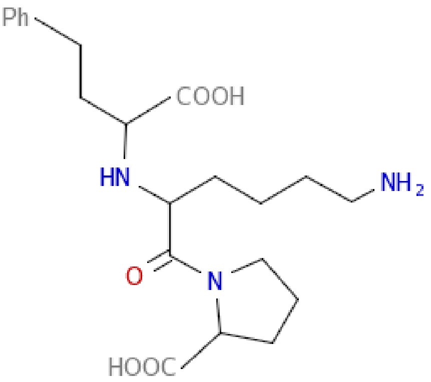

Lisinopril

SimilarityGroup: 5

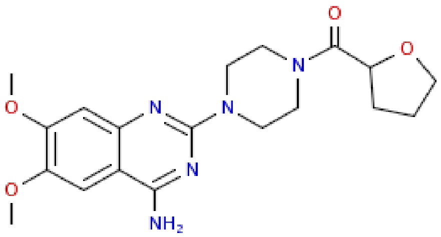

Terazosin

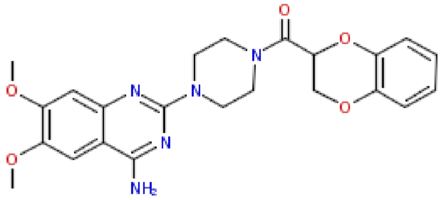

Doxazosin

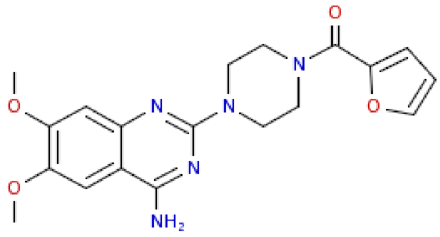

Prazosin

SimilarityGroup: 6

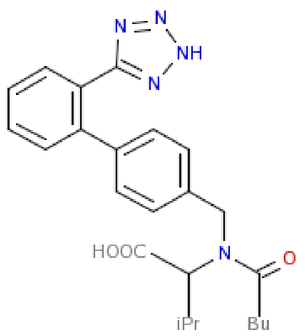

Valsartan

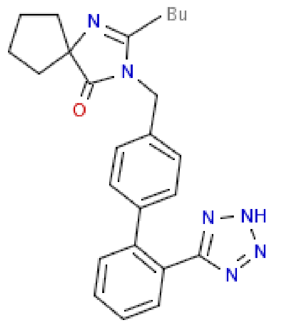

Irbesartan

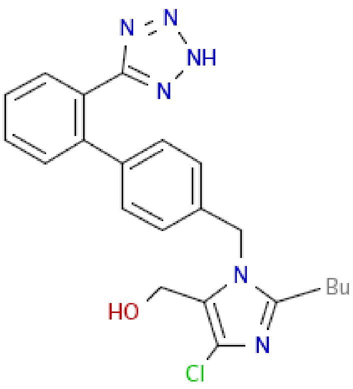

Losartan

SimilarityGroup: 7

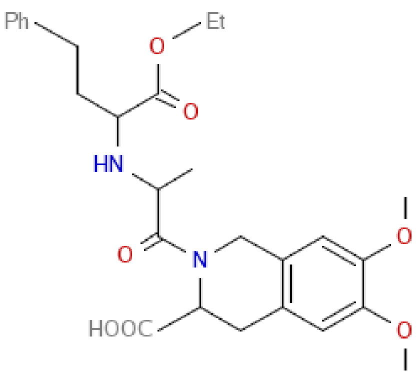

Moexipril

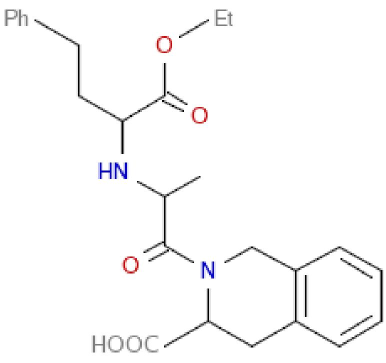

Quinapril

SimilarityGroup: Singletons

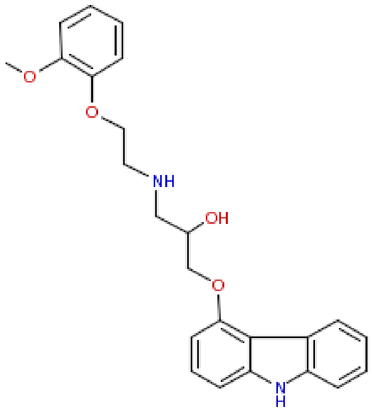

Carvedilol

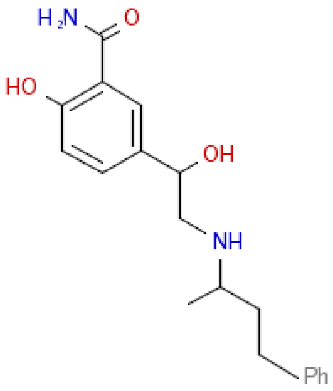

Labetalol

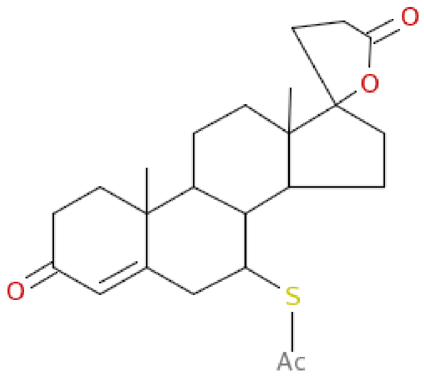

Spironolactone

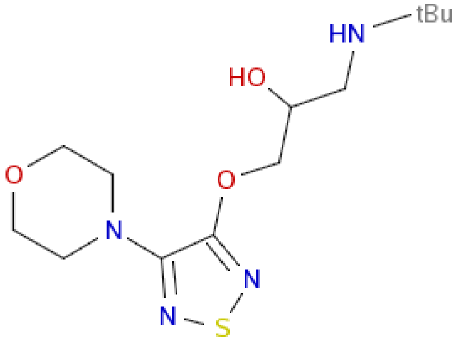

Timolol

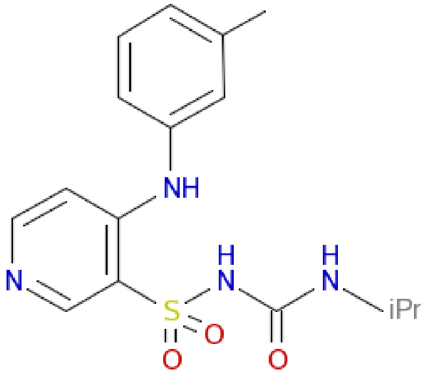

Torasemide

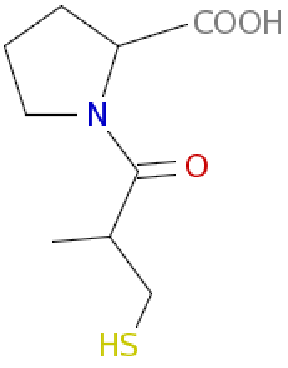

Captopril

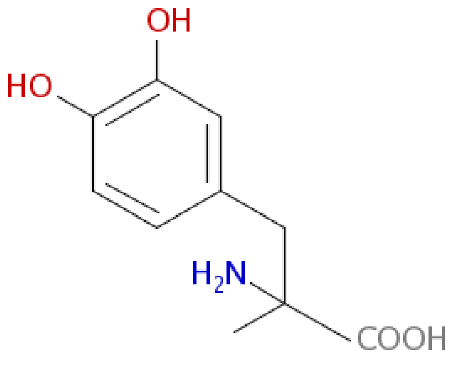

Methyldopa

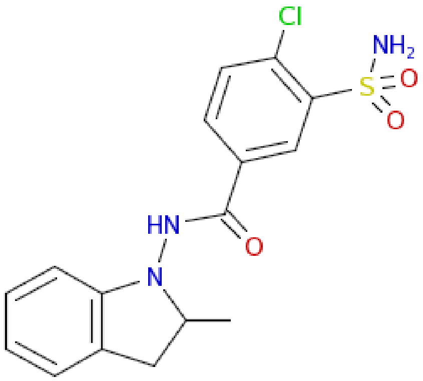

Indapamide

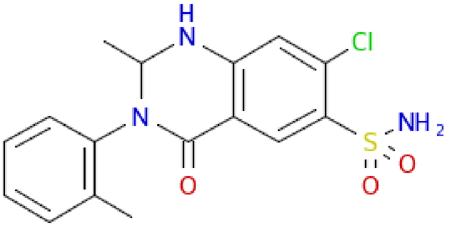

Metolazone

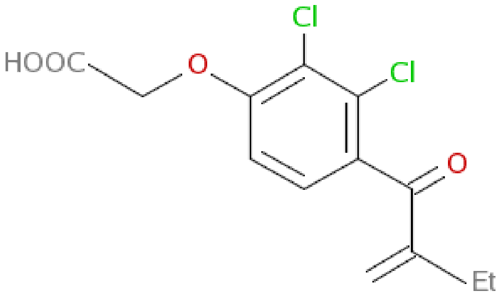

Ethacrynic acid

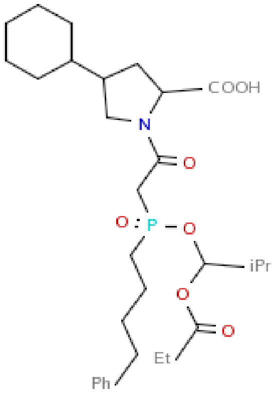

Fosinopril

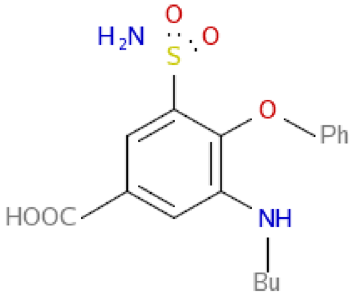

Bumetanide

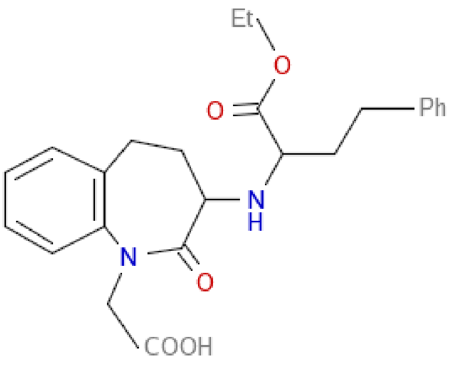

Benazepril

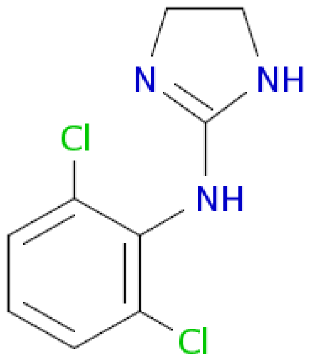

Clonidine

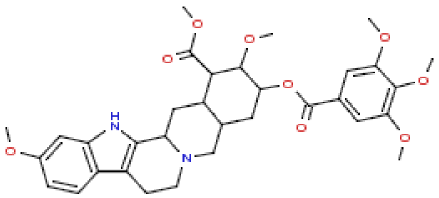

Reserpine

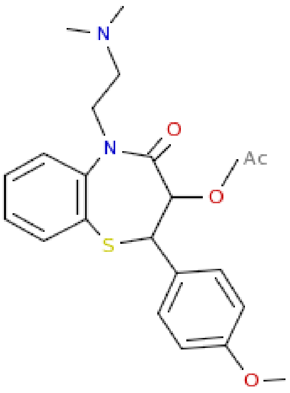

Diltiazem

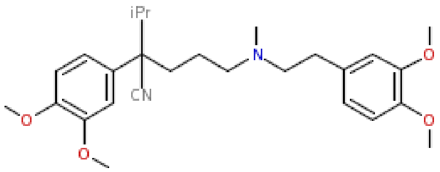

Verapamil

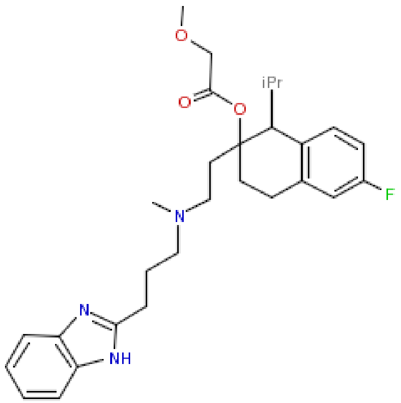

Mibefradil

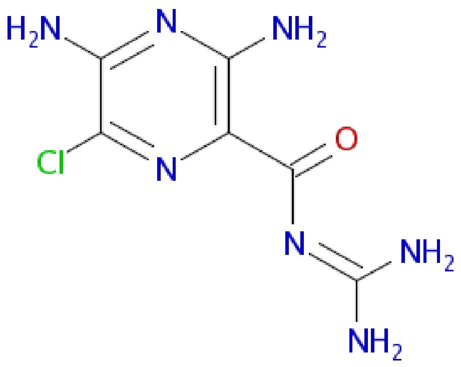

Amiloride

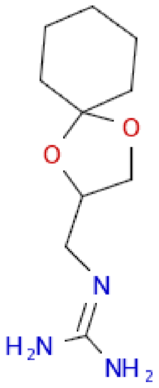

Guanadrel Sulfate

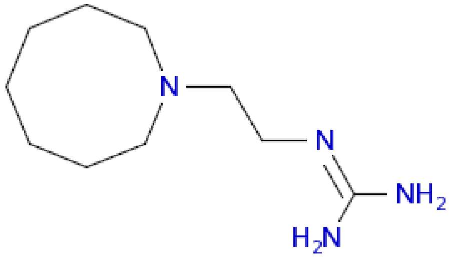

Guanethidine

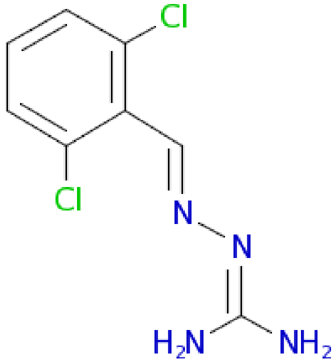

Guanabenz

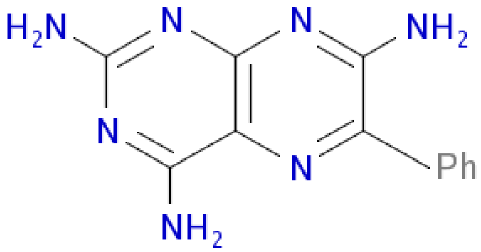

Triamterene

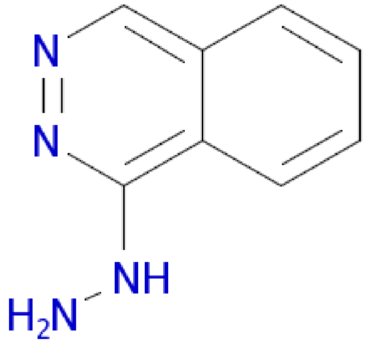

Hydralazine

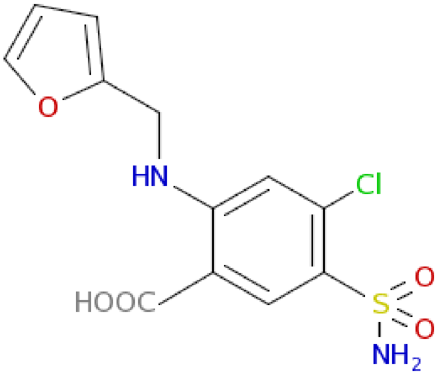

Furosemide

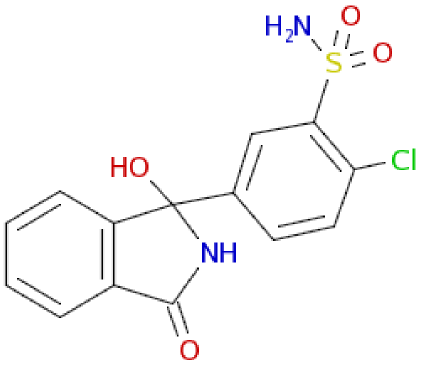

Chlorthalidone

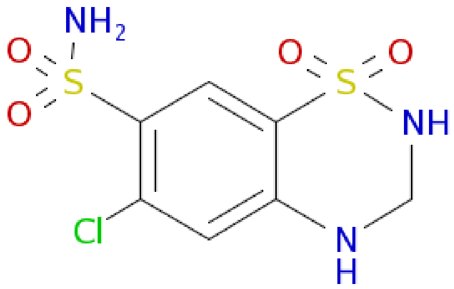

Hydrochlorothiazide
