# Supplementary material for: Exploiting large-scale drug-protein interaction information for computational drug repurposing
Source: BMC Bioinformatics. 2014 Jun 20;15:210. doi: 10.1186/1471-2105-15-210 (PMC4079911; doi:10.1186/1471-2105-15-210)
Supplement: Additional file 2: Figure S2 — HIV drugs grouped by molecular structure similarity. Molecular structure similarity clusters of the HIV drugs. [file 1471-2105-15-210-S2.pdf]

Figure S2. HIV drugs grouped by molecular structure similarity

SimilarityGroup: 1

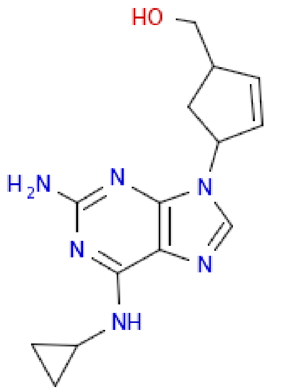

Abacavir

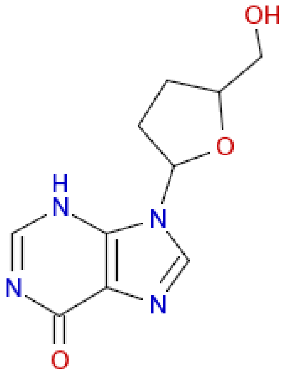

Didanosine

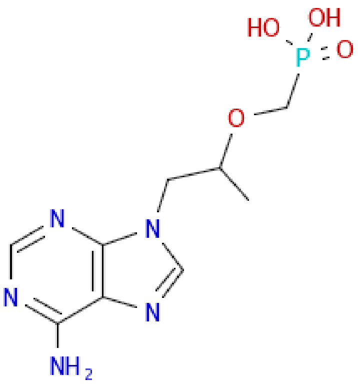

Tenofovir

SimilarityGroup: 2

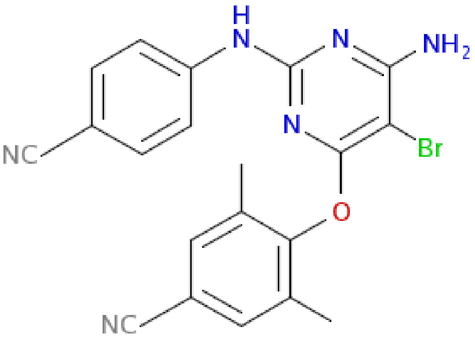

Etravirine

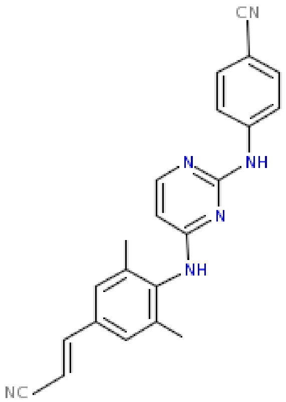

Rilpivirine

SimilarityGroup: 3

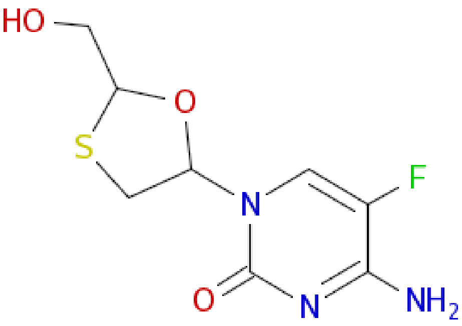

Emtricitabine

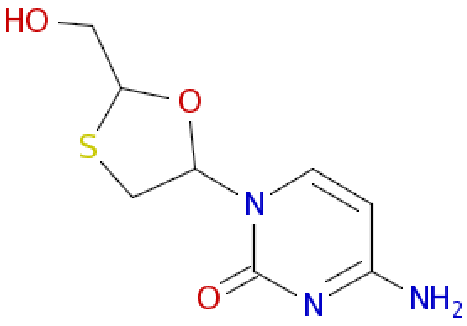

Lamivudine

SimilarityGroup: 4

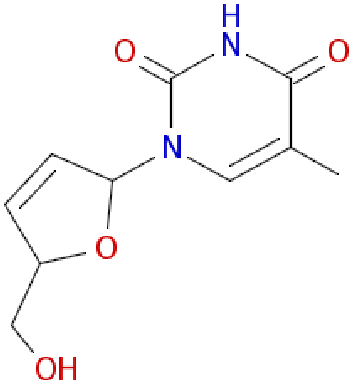

Stavudine

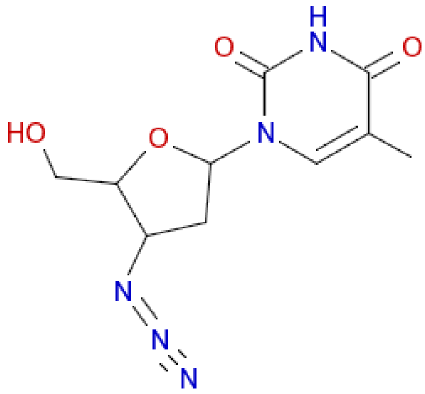

Zidovudine

SimilarityGroup: 5

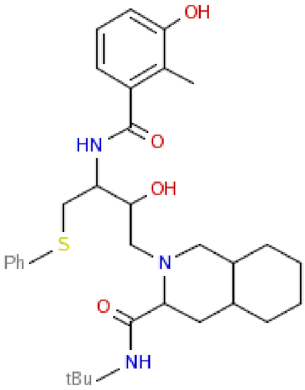

Nelfinavir

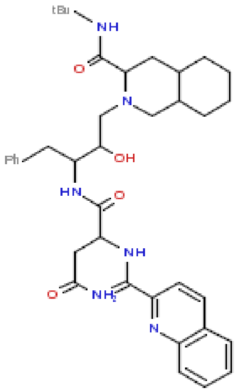

Saquinavir

SimilarityGroup: 6

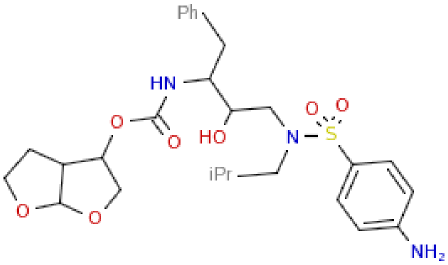

Darunavir

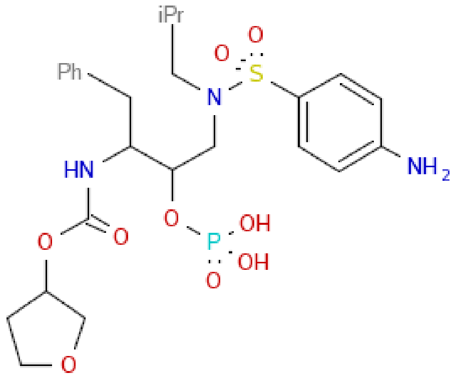

Fosamprenavir

SimilarityGroup: Singletons

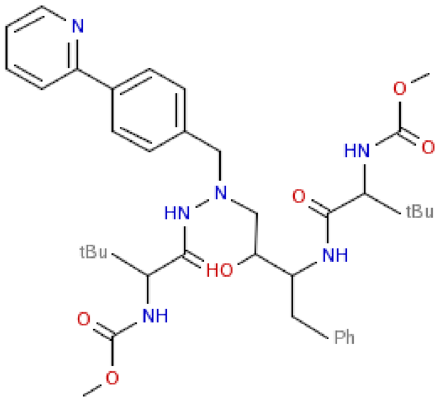

Atazanavir

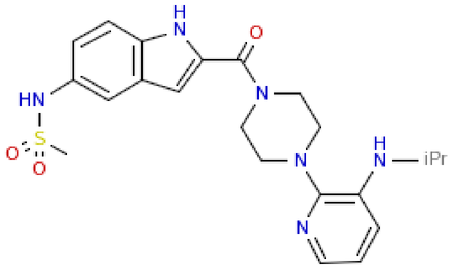

Delavirdine

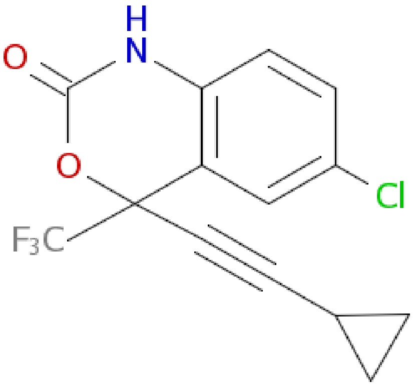

Efavirenz

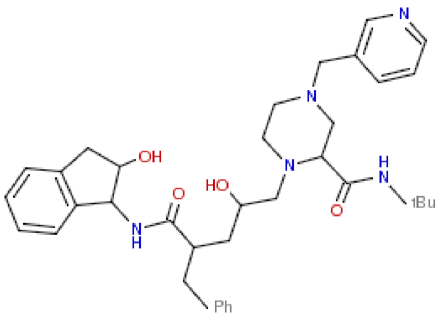

Indinavir

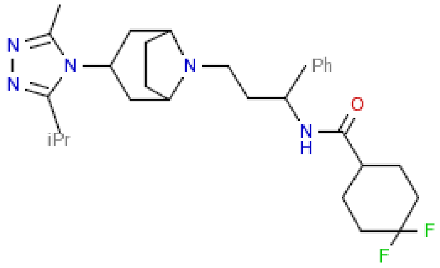

Maraviroc

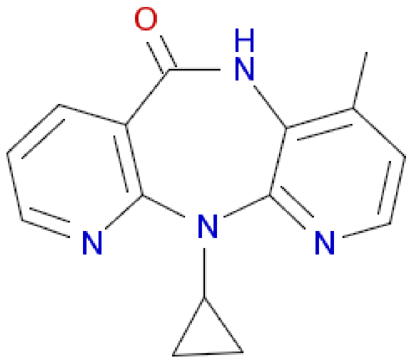

Nevirapine

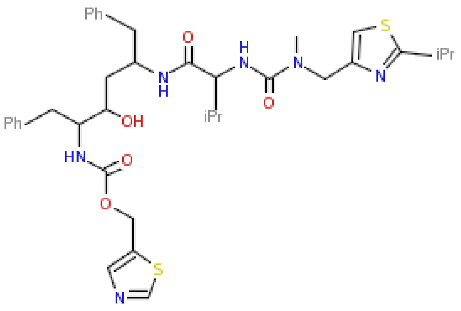

Ritonavir
