# Supplementary material for: Exploiting large-scale drug-protein interaction information for computational drug repurposing
Source: BMC Bioinformatics. 2014 Jun 20;15:210. doi: 10.1186/1471-2105-15-210 (PMC4079911; doi:10.1186/1471-2105-15-210)
Supplement: Additional file 3: Figure S3 — Malaria drugs grouped by molecular structure similarity. Molecular structure similarity clusters of the malaria drugs. [file 1471-2105-15-210-S3.pdf]

Figure S3. Malaria drugs grouped by molecular structure similarity

SimilarityGroup: 1

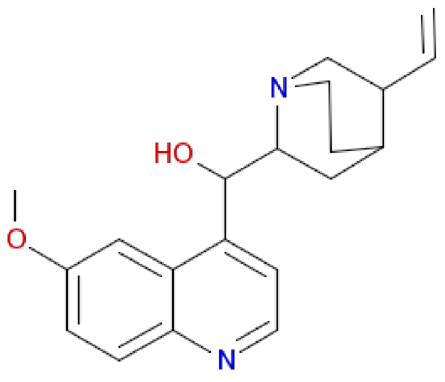

Quinine

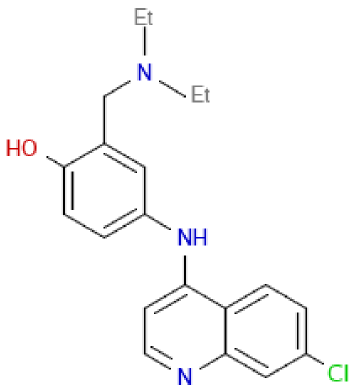

Amodiaquine

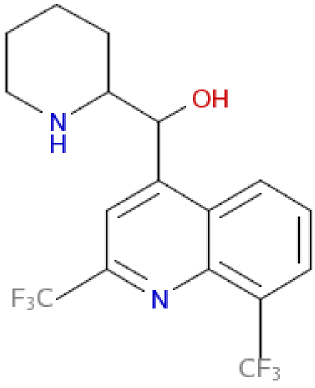

Mefloquine

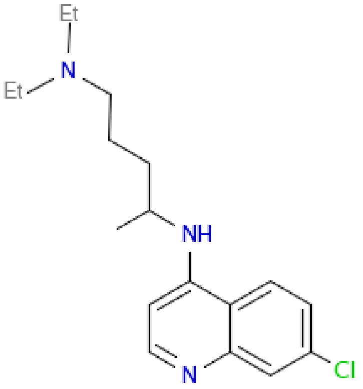

Chloroquine

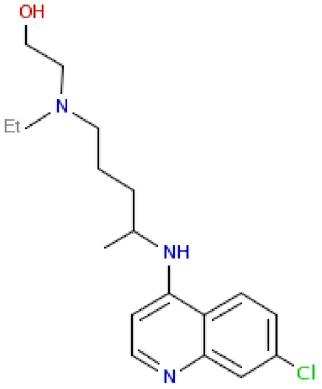

Hydroxychloroquine

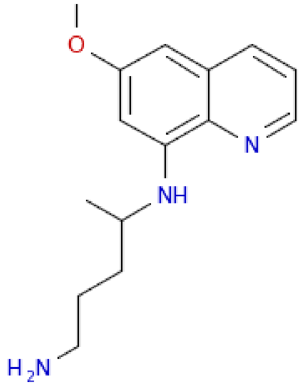

Primaquine

SimilarityGroup: Singleton

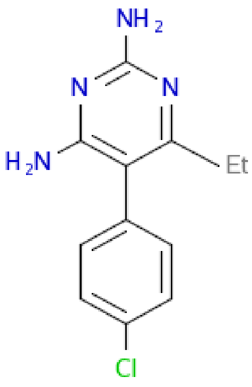

Pyrimethamine

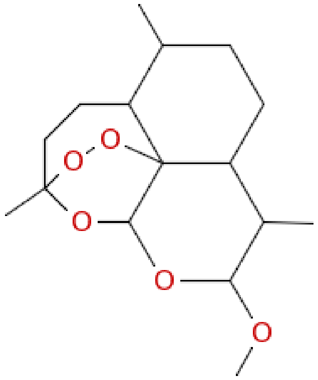

Artemether

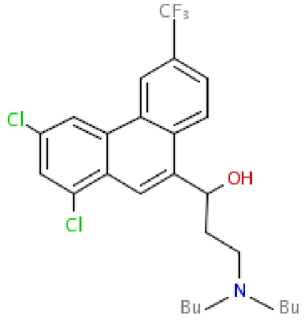

Halofantrine

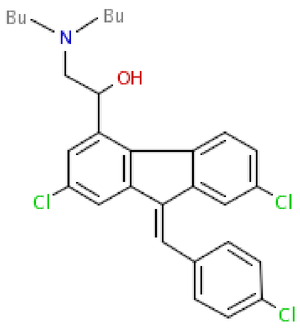

Lumefantrine

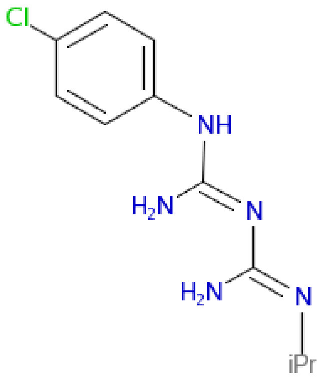

Proguanil
